
JOURNAL INFORMATION
==============================
NLM Title Abbreviation: Gastroenterol Rep (Oxf)
Journal ID: Gastroenterol Rep (Oxf)
NLM Journal ID: 101620508
Journal ID: gastro

Gastroenterology Report

EISSN: 2052-0034
Publisher: Oxford University Press

ARTICLE INFORMATION
==============================
PMCID: PMC5691613
DOI: 10.1093/gastro/gox038
Article ID: gox038
Article version: 1
Subjects: Chinese Abstracts

Chinese Abstracts – Volume 5 Issue 4*

* This article abstract was originally published in English. This translation has not been verified and should not be relied upon—it is provided for reference purposes only. The Publishers have not checked this translation and accept no liability for completeness or accuracy of this translation or the use of this translation for whatever purpose. This translation may be incomplete and inaccurate in whole or in part. If you need to rely upon a translation of this abstract, a professional human translator should be engaged to supply an accurate translation of the original English. When referencing articles from this journal, please always refer to the original English version, rather than a translated equivalent.

Print publication date: 2017 Nov
Electronic publication date: 2017 Nov 13
Volume: 5
Issue: 4
First page: C1
Last page: C3
Copyright: © The Author(s) 2017. Published by Oxford University Press and Sixth Affiliated Hospital of Sun Yat-Sen University
Copyright year: 2017
License: This is an Open Access article distributed under the terms of the Creative Commons Attribution License (http://creativecommons.org/licenses/by/4.0/), which permits unrestricted reuse, distribution, and reproduction in any medium, provided the original work is properly cited.
License URL: https://creativecommons.org/licenses/by/4.0/

Page count: 3

==============================
Gastroenterology Report, 2017, 1–3

doi: 10.1093/gastro/gox038

ABSTRACTS

### Chinese Abstracts – Volume 5 Issue 4*

### Review (综述)

### Diagnosis and therapy of esophageal squamous cell dysplasia and early esophageal squamous cell cancer
### 食管鳞状细胞不典型增生和早期食管鳞癌的诊断与治疗

[摘要] 食管癌主要包括鳞癌和腺癌，这两种性质的食管癌在多个方面存在差异。尽管食管腺癌发生率在西方国家持续升高， 但在世界范围内食管鳞癌仍然占多数。鳞状细胞不典型增生被认为是食管鳞癌的一种癌前病变，然而，其恶性转移的概率及进程尚不清楚，目前亦缺乏一个标准的处理规范。食管鳞癌若不能早期发现，预后较差，因此，早期诊断和早期治疗是一个趋势。诊断手段包括内镜超声、白光内镜以及生物学标志物等。早期诊断能使患者获益于内镜切除治疗。本文旨在探讨早期食管鳞状细胞病变的各种诊治方法，包括一些新的内镜治疗技术，并对这些不同的技术进行评价。

关键词：食管鳞癌；食管鳞状细胞不典型增生；内镜切除；射频消融

### Intestinal autotransplantation
### 肠道自体移植

[摘要] 大多数累及肠系膜上动脉根部和/或腹主动脉的腹部肿瘤难以通过传统的外科手术进行切除， 因为肠缺血时间有限且肿瘤区域难以完全暴露。体外手术后自体移植（IATx）是一种相对较新的外科技术，能在这些根治无望的情况下提供一个肿瘤完全切除的机会。本文旨在评估IATx的潜在适应证、手术技术及临床疗效。目前报道的IATx主要适应证包括侵犯肠系膜上血管的胰腺、肠系膜及腹膜后肿瘤。初步的结果显示， 对于严格筛选的病例， IATx可以获得满意的根治性切除。尽管围手术期并发症发生率和病死率相对较高，仍有部分患者可以获得长期存活，尤其是完全切除的良性或低度恶性肿瘤。而对于高度恶性的肿瘤尤其是胰腺导管癌，术后早期复发仍然是一大难题。总之，IATx可以使部分侵及肠系膜主要血管的腹部肿瘤得以完全切除。然而， 这种激进的术式具有相当高的手术风险，目前仅推荐在少数经验丰富的中心开展。另外，也需要配合其他的一些治疗措施以及辅助治疗，以提高IATx的疗效。

关键词：肠道自体移植；体外手术；胰腺癌；肠系膜肿瘤

### From genetics and epigenetics to the future of precision treatment for obesity
### 从遗传学和静观遗传学的角度展望肥胖症精准治疗的未来

[摘要] 肥胖症已经成了影响全球健康的主要问题。简单来说，肥胖症是由于能量摄取与消耗的失衡所导致的人体脂肪堆积。肥胖症的治疗包括纠正营养过剩、增加体力活动、药物治疗或外科手术。但每种治疗方法的疗效在不同个体中却不尽相同。随着肥胖症相关的遗传学和表观遗传学的研究进展， 临床上的个体化治疗或将实现。本文对肥胖症遗传学和表观遗传学的研究现状及其如何影响肥胖症的治疗效果进行了综述。由于减重手术是病态肥胖症最长期有效的治疗手段， 我们特别关注了遗传因素与减重手术临床疗效的关系。最后， 我们对肥胖症精准治疗的前景进行了展望。

关键词：病态肥胖症；遗传学；表观遗传学；减重手术

### Original Article (论著)

### Correlation of the Rutgeerts score and recurrence of Crohn’s disease in patients with end ileostomy
### Rutgeerts 评分与克罗恩病末端回肠造口术后复发的相关性

[摘要] 背景：克罗恩病（CD）患者术后（包括末端回肠造口术）会出现复发。对于回结肠切除术患者，术后可以通过回结肠镜进行Rutgeerts评分，从而预测术后CD的临床复发。而通过回肠造口进行的回肠镜评估对于术后CD复发的预测价值， 目前尚未见报道。本研究旨在评估回肠镜下Rutgeerts评分对于CD患者末端回肠造口术后疾病复发的预测价值。

方法：共有73例行末端回肠造口术的CD患者纳入研究， 所有入组病例术后均在本中心接受了至少2次的回肠镜评估。基于Rutgeerts评分对新末端回肠的黏膜炎症程度进行分级。主要结局指标是由于疾病复发需要行内镜扩张治疗或再次手术治疗，次要结局指标是CD相关住院治疗和需要升级CD治疗药物。

结果：本组病例CD发病至行末端回肠造口术的中位间期为9年（四位分间距：413年），造口术至首次回肠镜检查的中位间期为28月（四位分间距：1193月）。对靠近造口处的新末端回肠进行Rutgeerts评分，并据此将患者分为两组：Rutgeerts评分0分者为正常评分组（25例），≥1分者

---

*This article abstract was originally published in English. This translation has not been verified and should not be relied upon—it is provided for reference purposes only. The Publishers have not checked this translation and accept no liability for completeness or accuracy of this translation or the use of this translation for whatever purpose. This translation may be incomplete and inaccurate in whole or in part. If you need to rely upon a translation of this abstract, a professional human translator should be engaged to supply an accurate translation of the original English. When referencing articles from this journal, please always refer to the original English version, rather than a translated equivalent.
本文摘要最初是以英文发表。中文摘译未获认证，谨供参考。出版社没有对中文摘译进行核验，不负责译文的完整性和准确性，也不会将其用以其他目的。中文摘译可能会出现部分不完整或不准确的情况。如果你需要准确获悉摘要，最好能请一位专业译者针对英文原文进行翻译。阅读杂志文章，请参考英文原文，不推荐中文翻译。

为异常评分组（48例）。异常评分组患者疾病复发（92% *vs* 27%）、需要行内镜扩张治疗（40% *vs* 10%）、再次肠道手术（68% *vs* 15%）、住院治疗（80% *vs* 23%）以及CD治疗药物升级的概率高于正常评分组。事件发生时间（time-to-event）分析显示，对手术至首次内镜评估的时间、疾病病程和患者性别等因素进行调整后， 异常评分组患者需要行内镜扩张（HR=1.5，95%CI：1.09–1.9）、再次肠道手术（HR=1.5，95%CI：1.2–1.8）和住院治疗（HR=1.3，95%CI：1.1–1.6）的风险显著增高。多因素分析显示，对手术至初次内镜评估的时间和患者年龄进行调整后，异常评分组患者需要升级CD治疗药物的风险亦明显增高 （OR=5.3， 95%CI：1.7–16.5；*P*=0.004）。

结论：Rutgeerts评分可用以预测末端回肠造口患者的CD复发。回肠镜评估的高Rutgeerts评分患者预后不良。基于回肠镜的Rutgeerts评分是末端回肠造口术后疾病监测的有效工具。

关键词：克罗恩病；Rutgeerts评分；回肠镜；回肠造口术；复发

### Endoscopic retrograde cholangiopancreatography with ampullary biopsy vs ERCP alone: a matched-pairs controlled evaluation of outcomes and complications

### 内镜逆行性胆胰管造影（ERCP）联合壶腹活检与单纯ERCP诊断收益及并发症的比较：配对病例对照研究

[摘要] 背景与目的：Vater壶腹活检可对壶腹腺瘤和可疑的壶腹肿瘤进行评估， 并对自身免疫性胰腺炎进行免疫组化检测。壶腹活检通常是在内镜逆行性胆胰管造影（ERCP）的同时进行。ERCP存在一定的并发症，壶腹活检是否会增加ERCP术后并发症发生率需要病例对照研究的验证。

方法：对行ERCP伴或不伴壶腹活检的病例进行配对病例对照研究。回顾性分析在一家三甲医院行ERCP和壶腹活检的成人病例，并按操作复杂程度匹配一组未作壶腹活检的ERCP病例作为对照。

结果：在159例进行壶腹活检的病例中，通过ERCP完成壶腹活检的54例病例纳入研究组。根据美国胃肠内镜协会（ASGE）的操作复杂性分级进行1:1匹配， 54例未行壳腹活检的ERCP病例纳入对照组。所有病例均无Oddi括约肌功能异常。壶腹活检为75.9%的病例提供了诊断，包括12例腺瘤、5例腺癌和1例导管内乳头状黏液肿瘤。行壶腹活检的研究组术后总并发症（包括严重并发症和轻微并发症）发生率为9.3%，与未行壶腹活检的对照组相当（9.3%，*P* = 0.99）。两组病例ERCP术后胰腺炎发生率的差异亦无统计学意义（5.6% *vs* 3.7%，*P* = 0.6）。多因素分析显示，高龄和胰管操作是ERCP并发症的独立危险因素， 而壶腹活检与ERCP并发症的发生无关。

结论：在行ERCP进行壶腹活检可以获得一个较高的诊断收益， 而且并不会增加ERCP术后并发症和胰腺炎的发生率。

关键词：内镜逆行性肝胰管造影；壶腹活检；并发症

### Hepatic paragonimiasis: a single-center retrospective analysis of 32 cases in Mainland China

### 肝吸虫病：中国大陆单中心32例回顾性分析

[摘要] 背景：肺和胸腔是吸虫病的主要受累器官，此外，中枢神经系统也时有感染。肝是异位吸虫病极少累及的器官。由于肝吸虫病的症状和检查结果不典型，该病在临床上常被误诊。

方法：2008年2月至2015年3月间， 本中心收治了32例经超声和CT发现的肝肿块患者，经多项进一步检查，肿块来源仍无法明确。我们最终行外科手术切除并将组织标本送病理检查。我们对这些病例的临床资料进行分析，并对疾病特征、诊断策略及治疗经验进行了总结。此外，我们还对这些病伤残 进行了612月的随访。

结果：所有病例均行半肝或部分肝切除术并最终经病理诊断为肝吸虫病。术后均恢复良好，症状显著改善，无一例复发。出院前肝功能均达到正常水平，影像学检查亦未发现任何其他肝脏病变。

结论：基于临床表现、实验室检查及影像学检查难以诊断肝吸虫病，组织标本病理检查是惟一确诊肝吸虫病的手段。外科手术不仅可以切除病变， 而且可以提供病理标本。本研究是至今最大例数的肝吸虫病报道，但手术疗效需进一步研究和更长期的随访。

关键词：肝吸虫病；寄生虫病；诊断策略；肝切除术

### Rates of hospitalization among African American and Caucasian American patients with Crohn's disease seen at a tertiary care center

### 非洲裔美国人与白种美国人克罗恩病住院率：一家炎性肠病三级转诊中心的数据

[摘要] 背景：对于非洲裔美国人与白种美国人克罗恩病（CD）疾病进程和临床结局是否存在差异， 目前尚缺乏明确的结论。我们旨在探讨非洲裔美国人与白种美国人相比，是否有更大的概率会因CD相关并发症而接受住院治疗。

方法：我们进行了一项回顾性队列研究，纳入了2000—2013年间我们中心（炎性肠病三级转诊中心）的909例克罗恩病患者，其中非洲裔美国人207例，白种美国人702例。我们统计了这两个种族因CD并发症的住院率。采用零膨胀Poisson回归模型来估计CD相关住院的粗风险比（RR）和多因素校正后的RR。多因素模型中的校正因素包括年龄、性别、CD病程、吸烟史和CD治疗史。

结果：非洲裔美国人的累积CD相关住院率高于白种美国人 （395.6/1000 *vs* 230.4/1000）。比较非洲裔美国人与白种美国人CD相关住院率，未校正和多因素校正后的RR值分别为1.59 （95%CI：1.10–2.29；*P*=0.01）和1.44（95%CI：1.02–2.03；*P*=0.04）。

结论：从一个炎性肠病三级转诊中心来看，非洲裔美国人与白种美国人相比，CD相关住院率更高。后续应进一步评估社会经济地位和生物学标志物是否可以解释非洲裔美国人较高的住院率。

关键词：克罗恩病；种族差异；非洲裔美国人；白种美国人

### Predictors of acute gastrointestinal bleeding in diabetic ketoacidosis: a retrospective observational study in minority population

### 糖尿病酮症酸中毒患者发生胃肠道出血的预测因素：一项回顾性的观察研究

[摘要] 背景 酮症酸中毒（DKA）是糖尿病一种常见的急性并发症，需要积极的药物治疗。我们旨在研究DKA患者急性胃肠道出血（AGIB）和急性上消化道出血（AUGIB）的发生率及其相关危险因素。

方法：我们对本中心2010年1月至2015年12月间收治的DKA病例进行回顾性分析， 通过电子病历库收集其人口统计学、临床、实验室、内镜及住院资料。根据有无发生胃肠道出血将患者分为两组。

结果：共计234例DKA患者纳入研究，其中27例（11.5%）有AGIB的记录。除2例直肠出血和3例隐匿性出血外， 22例 （9.4%） 表现为呕血。AGIB组与非AGIB组患者年龄、性别和种族的分布无显著差异。两组患者电解质水平、阴离子间隙、pH值及糖化血红蛋白HbA1c的差异亦无统计学意义。然而， AGIB组患者初始血糖水平显著高于非AGIB组 （738 mg/dL *vs* 613 mg/dL，*P*=0.014）。AGIB组患者病死率也有所升高（7.4% *vs* 4.8%），尽管差异未达到统计学意义。

结论：初始高血糖水平会增加DKA患者发生AGIB的风险。我们还注意到发生AGIB的DKA患者病死率增加， 尽管这个增加未达到统计学意义。临床上应更加积极地纠正DKA患者的血糖水平以降低AGIB发生率，从而降低住院病死率。

关键词：糖尿病酮症酸中毒；胃肠道出血；病死率

### Preoperative hypoalbuminemia is associated with an increased risk for intra-abdominal septic complications after primary anastomosis for Crohn's disease

### 术前低蛋白血症会增加克罗恩病一期肠吻合术后腹腔内感染性并发症的发生风险

[摘要] 目的：本研究旨在探讨术前低蛋白血症对克罗恩病（CD）患者一期肠吻合术后发生腹腔内感染性并发症（IASCs）的影响。

方法：2007—2015年期间所有行肠切除一期肠吻合手术的CD患者纳入研究。采用单因素和多因素分析评估术前低蛋白血症（白蛋白<30 g/L）与术后IASCs发生风险的关系。

结果：共计124例患者纳入研究, 其中117例 (94.4%) 可以获得术前白蛋白水平的数据。13例 (11.7%) 患者术前合并低蛋白血症。相较之下, 术前低蛋白血症患者CD诊断至手术的间期更长 ($P$=0.012)，术前服用5氨基水杨酸的比例更高 ($P$=0.013)，术中发现小肠梗阻的比例更高 ($P$=0.015)。共有24例 (19.4%) 患者术后出现IASCs。单因素分析显示, 术前低蛋白血症患者术后IASCs风险更高 ($P$=0.012)。多因素分析证实, 术前低蛋白血症是术后IASCs的独立危险因素 (OR=4.67, 95%CI: 1.28-17.04, $P$=0.02)。将术前白蛋白水平作为一个连续型变量进行多因素分析, 亦可得到相同的结果 ($P$=0.019)。

结论：对于行肠切除一期肠吻合的CD患者, 术前低蛋白血症提示术后IASCs发生风险增高。改善术前营养状态或可降低术后IASCs的发生风险。

关键词：克罗恩病；低蛋白血症；腹腔内感染性并发症；危险因素

### Utility of Doppler ultrasonography for predicting improvement of platelet count after transjugular intrahepatic portosystemic shunt

### 应用多普勒超声预测经颈静脉肝内门体静脉分流术后血小板计数的改善

[摘要] 目的：经颈静脉肝内门体静脉分流术 (TIPS) 后门静脉压力梯度 (PPG) <12 mmHg被认为是血小板升高的惟一预测指标。按照Bernoulli方程, 多普勒超声检测的血流速率与压力梯度密切相关, 因此, 我们采用这一指标来预测TIPS术后血小板计数的升高。

方法：共计161例行TIPS手术的连续肝硬化患者连续纳入本回顾性研究。分别于TIPS术前、术后1周、术后1月检测血小板计数。有临床意义的血小板减少定义为血小板计数 ≤ 100 000。检测TIPS手术前后PPG水平。使用多普勒超声检测TIPS支架近、远端的血流速率, 将血流速率的差异定义为血流速率梯度 (FVG)，其正常值为 ≤ 100 cm/s。

结果：在121例术前血小板减少的患者中, 90例PPG ≤ 12 mmHg, 其术后1周和1月平均血小板计数分别升高了25.7 × $10^3$ ($P$=0.028) 和35.0 × $10^3$ ($P$=0.015)；而PPG > 12 mmHg的31例患者术后血小板计数无显著改变 ($P$=0.098和$P$=0.075)。FVG ≤ 100 cm/s (95例) 与 > 100 cm/s (26例) 患者术后1周血小板计数分别升高了37.0 × $10^3$ ($P$=0.005) 和11.0 × $10^3$ ($P$=0.07)，术后1月分别升高了17.0 × $10^3$ ($P$=0.01) 和5.2 × $10^3$ ($P$=0.21)。

结论：FVG > 100 cm/s的肝硬化患者TIPS术后血小板计数不会显著升高。另一方面, 正常的TIPS功能 (FVG ≤ 100 cm/s或PPG ≤ 12 mmHg) 提示TIPS术后血小板计数明显升高。

关键词：肝硬化；经颈静脉肝内门体静脉分流术；血小板计数；多普勒超声

### Case Report (病例报告)

### The missing appendix: a case report of appendiceal intussusception at the site of colonic mullerianosis

### 消失的阑尾：结肠子宫内膜异位处阑尾套叠一例报告

### An uncommon source of upper gastrointestinal bleeding: epiphrenic esophageal diverticulum

### 上消化道出血的一种罕见原因：膈上食管憩室

### *Mycobacterium avium* subspecies *hominissuis* in Crohn's disease: a case report

### 克罗恩病患者中培养出鸟型分支杆菌*hominissuis*亚种一例报告

### Hyperbaric oxygen therapy for chronic antibiotic-refractory ischemic pouchitis

### 高压氧治疗慢性抗生素难治性缺血性储袋炎
